# The Combination Usage of Dexamethasone and Prednisolone Versus Prednisolone Monotherapy for Inflammation and Intraocular Pressure After Cataract Surgery

**DOI:** 10.7759/cureus.76790

**Published:** 2025-01-02

**Authors:** Chia-Yi Lee, Shun-Fa Yang, Chin-Te Huang, Jing-Yang Huang, Chao Kai Chang

**Affiliations:** 1 Institute of Medicine, Chung Shan Medical University, Taichung, TWN; 2 Department of Medical Research, Chung Shan Medical University Hospital, Taichung, TWN; 3 Department of Ophthalmology, Chung Shan Medical University Hospital, Taichung, TWN; 4 Department of Ophthalmology, Nobel Eye Institute, Taipei, TWN

**Keywords:** cataract surgery, dexamethasone, inflammation, intraocular pressure, prednisolone

## Abstract

Purpose

The purpose of this study is to survey the anti-inflammatory effect of combined preoperative dexamethasone and postoperative prednisolone therapy and postoperative prednisolone therapy for cataract surgery. The intraocular pressure (IOP) change between groups was also examined.

Methods

A retrospective cohort study was practiced, and individuals who received cataract surgery were enrolled and categorized according to the anti-inflammation therapy. A total of 62 and 108 eyes were included in the combined and single groups, respectively. The primary outcomes were ocular hypertension and anterior chamber (AC) inflammation. The independent t-test and generalized linear model were used for the statistical analysis.

Results

There were 12 and 16 episodes of ocular hypertension in the combined and single groups, and the incidence of ocular hypertension was similar between groups (P = 0.203). In addition, there were four and seven AC inflammation events in the combined and single groups, and the incidences of AC inflammation were similar between groups (P = 0.335). Old age was correlated to a higher risk of developing ocular hypertension in the combined and single groups (both P < 0.05). Besides, higher myopia is related to higher ocular hypertension risk in the combined group (P = 0.038). Preoperative dry eye disease (DED) was associated with higher AC inflammation risk in the combined and single groups (both P < 0.05). Also, the high myopia was related to higher AC inflammation risk in the two groups (both P < 0.05).

Conclusion

The combination therapy of dexamethasone and prednisolone showed similar anti-inflammatory effects. The IOP changes between groups were similar.

## Introduction

Cataract is a widespread ocular disease that causes impaired visual acuity, and surgery is the only effective intervention to manage the formed cataract [1]. Phacoemulsification with intraocular lens implantation (IOL) is currently the main type of cataract surgery that features small wounds and faster postoperative recovery [2]. The general postoperative outcome for cataract surgery is fair, in which a postoperative visual acuity of more than 20/40 on the Snellen chart is not difficult to achieve [3,4]. In addition, refractive correction become an important issue for cataract surgery in recent years, and the postoperative refractive error was lower than ±1.00 diopter (D) in most cases [5,6].

Although surgical success is common in patients who receive cataract surgery, complications can still develop after the cataract surgery [7]. The severe complications after cataract surgery include infectious endophthalmitis, pseudophakic bullous keratopathy, and retinal detachment [8-11]. Besides, ocular hypertension is also a prominent postoperative complication of cataract surgery, which can result from the presence of diabetes mellitus or residual cortex [12]. Intraocular inflammation is another post-cataract surgery complication that results from the intraoperative damage of ocular structure and occurs in nearly all ocular surgeries [13]. If left untreated, the intraocular inflammation may cause cystoid macular edema and visual loss [14].

For the management of postoperative inflammation, the application of anti-inflammatory medication is the most common intervention [15]. Topical prednisolone has been applied to control postoperative inflammation, while intraocular pressure (IOP) status should be checked [16,17]. Moreover, intraoperative dexamethasone has been utilized in patients with cataract surgery with fair inflammation control and IOP change [18,19]. Still, the dexamethasone implant may cause a floater sensation, and being too invasive to some patients, the preoperative usage of dexamethasone may be another way that needs to be investigated.

As a result, the objective of this study is to evaluate the effectiveness of preoperative dexamethasone and postoperative prednisolone on postoperative inflammation compared to postoperative prednisolone monotherapy. Besides, the fluctuation of IOP after cataract surgery between the two managements was also evaluated.

## Materials and methods

Ethics declaration

This study stands by the Declaration of Helsinki in 1964 and the later amendments. This study was accepted by the Institutional Review Board of the National Changhua University of Education (code: NCUEREC-113-057, date of approval: 07/03/2024). The need for written informed consent obtainment was discarded by the Institutional Review Board of the National Changhua University of Education.

Subject selection

A retrospective cohort study was completed at the Nobel Eye Institute, which is a joint clinical institution with more than 20 branches throughout the Taiwan region. The individuals with the following characteristics were included in this study: (1) diagnosis of senile cataract in any clinic of Nobel Eye Institute, (2) their cataract surgery was done in any clinic of Nobel Eye Institute, (3) follow-up at any clinic of Nobel Eye Institute for more than three months, and (4) age older than 55 years. To better control the ocular condition of our patients, these exclusion criteria were used: (1) the emergence of corneal ulcer, corneal erosion, or extremely severe dry eye disease (DED) with diffuse punctate keratitis before the cataract surgery, (2) the emergence of uveitis within six months before the cataract surgery, (3) the emergence of uncontrolled glaucoma within six months before the cataract surgery, (4) the emergence of proliferative diabetic retinopathy and before the cataract surgery, (5) the emergence of severe blepharitis within six months before the cataract surgery, and (6) the emergence of lagophthalmos before the cataract surgery. Then, the patients were separated into a combined group and a single group according to whether they used preoperative dexamethasone or not. The usage of dexamethasone or not is according to the presence of a previous severe blepharitis history (six months earlier than the cataract surgery). Also, only the first eye of each patient that received cataract surgery was selected in this study. Finally, a total of 62 and 108 eyes were included in the combined group and single group, respectively.

Usage of anti-inflammatory agent

All the cataract surgery was accomplished by one ophthalmologist (C.-Y.L.) with one phacoemulsification machine (Quatera, Carl Zeiss, Jena, Germany). After the surgery, the 1% prednisolone acetate was instilled four times a day for one week, then switched to fluorometholone two times a day for another three weeks in the single group. In the combined group, the postoperative anti-inflammatory medications were the same as the single group, but the 1% dexamethasone phosphate was applied for one week before the cataract surgery. For controlling IOP, the anti-glaucomatous medications would be utilized if the IOP was higher than 21 mmHg in the postoperative period.

Ophthalmic examination

All the patients received the preoperative and postoperative examinations with the same devices in this study. About the preoperative exam, the uncorrected distance visual acuity (UDVA) and corrected distance visual acuity (CDVA) were measured using the Snellen chart and transferred into the LogMAR unit. The cycloplegia refraction was obtained with the assistance of an autorefractor (KR-8900, Topcon, Tokyo, Japan), and the spherical equivalent (SE) was defined as the sphere power plus half of the cylinder power. The IOP was measured by pneumatic tonometry (NT-530, Nidek Co. Ltd., Gamagori, Japan), while corneal astigmatism and biometric data were obtained via the topographic machine (TMS-5, Tomey Corporation, Nagoya, Japan) and biometry machine (IOL Master 700, Carl Zeiss, Jena, Germany), respectively. In addition, the preoperative tear break-up time (TBUT) was measured using fluorescein dye instillation and the slit-lamp biomicroscope. Also, the Schirmer I test with topical anesthesia was performed before the cataract surgery. For the postoperative exam, the UDVA, IOP, SE, and the presence of anterior chamber (AC) inflammation were obtained. The presence of AC inflammation was according to the criteria of the Standardization of Uveitis Nomenclature (SUN) workshop [20], and a grade of flare and cell higher than 2+ was defined as AC inflammation in this study. The data from one day, one week, one month, and three months after the cataract surgery were collected and analyzed.

Statistical analysis

The SPSS version 20.0 (IBM SPSS Statistics for Windows, IBM Corp., Armonk, NY) was used for all statistical analyses in this study. The statistical power for this study was 0.92 with a 0.05 alpha value, and the medium effect size was calculated by G∗Power version 3.1.9.2 (Heinrich Heine Universität, Düsseldorf, Germany). The Shapiro-Wilk test was used to confirm all the data normality, and normal distributions of all data in this study were found (all P > 0.05). The descriptive analysis was used to present the demography, preoperative visual acuity, refractive status, and DED status of the two groups, and independent t-test and chi-square test were used to compare the above parameters between groups based on the characteristics of the parameters. Then, the independent t-test was used to analyze the postoperative UDVA, SE, and IOP between the combined and single groups. In the following step, the generalized linear model was used to compare the risk of ocular hypertension and AC inflammation between groups, and the adjusted odds ratio (aOR) with a 95% confidence interval (CI) was calculated. The effect of age and sex was put into the generalized linear model. Besides, the preoperative predisposing factors for ocular hypertension and AC inflammation in the two groups were analyzed by the generalized linear model again. The preoperative predisposing factors put in the analysis include old age (> 65 years), male sex, preoperative DED (TBUT < 10 seconds or Schirmer test < 5 mm), presence of systemic disease, and high myopia (SE < -6.00 D). The statistical significance was represented as P < 0.05, and a P-value lower than 0.001 was represented as P < 0.001.

## Results

The initial characteristics of the two populations are demonstrated in Table 1. The mean age was 62.75 ± 6.61 and 62.13 ± 7.32 years in the combined group and single group, which were without significant differences (P = 0.583). The distribution of sex, systemic diseases, or laterality between the combined group and single group also demonstrated insignificant differences (all P > 0.05). Regarding the preoperative cycloplegia refraction and DED indexes, all these indexes showed similar values between the combined group and single group (all P > 0.05) (Table 1).

**Table 1:** The baseline features of the study population CDVA: corrected distance visual acuity, D: diopter, N: number, SE: spherical equivalent, TBUT: tear break-up time, UDVA: uncorrected distance visual acuity The statistical significance was defined as P < 0.05.

Feature	Combined group (N = 62)	Single group (N = 108)	P
Age (years)	62.75 ± 6.61	62.13 ± 7.32	0.583
Sex (male:female)	34:28	53:55	0.286
Laterality (right:left)	30:32	54:54	0.483
Disease	-	-	0.744
Hypertension	6	5	-
Diabetes mellitus	7	10	-
Autoimmune disease	2	4	-
Other	1	2	-
UDVA (LogMAR)	0.63 ± 0.25	0.58 ± 0.39	0.367
CDVA (LogMAR)	0.42 ± 0.17	0.45 ± 0.20	0.323
Cycloplegia refraction (D)	-	-	-
Sphere	-1.89 ± 2.94	-1.78 ± 3.24	0.826
Cylinder	-0.94 ± 0.43	-0.85 ± 0.37	0.152
SE	-2.36 ± 2.52	-2.21 ± 2.73	0.724
Schirmer test (second)	11.02 ± 2.26	11.65 ± 2.49	0.103
TBUT (second)	9.79 ± 1.95	10.33 ± 2.16	0.106

The UDVA one day postoperatively was 0.22 ± 0.13 and 0.23 ± 0.15 in the combined group and single group (P = 0.661). Three months postoperatively, the UDVA was significantly better in the single group than the combined group (0.04 ± 0.03 versus 0.03 ± 0.03, P = 0.038). About the postoperative refraction, all the SEs were similar between the combined group and single group throughout the follow-up period (all P > 0.05). The IOPs one day postoperatively (19.56 ± 5.53 versus 17.72 ± 4.37, P = 0.018) and one week postoperatively (17.31 ± 3.82 versus 15.23 ± 3.19, P < 0.001) were significantly higher in the combined group than the single group, but the IOP difference became insignificant in the following period (both P > 0.05) (Table 2). There were 12 and 16 episodes of ocular hypertension in the combined group and single group, respectively, and the incidence of ocular hypertension was similar between groups (aOR: 1.10, 95% CI: 0.96-1.25, P = 0.203) (Table 3). In addition, there were four and seven AC inflammation events in the combined group and single group, respectively, and the incidences of AC inflammation were similar between groups (aOR: 0.97, 95% CI: 0.92-1.02, P = 0.335) (Table 3). Of note, no cystoid macular edema was observed in both the combined group and single group.

**Table 2:** Postoperative visual and refractive outcomes between the two groups D: diopter, IOP: intraocular pressure, N: number, SE: spherical equivalent, UDVA: uncorrected distance visual acuity * denotes significant differences between groups. The statistical significance was defined as P < 0.05.

Outcome	Combined group	Single group	P
UDVA (LogMAR)			
One day	0.22 ± 0.13	0.23 ± 0.15	0.661
One week	0.12 ± 0.07	0.11 ± 0.08	0.413
One month	0.04 ± 0.05	0.05 ± 0.03	0.105
Three months	0.04 ± 0.03	0.03 ± 0.03	0.038*
SE (D)			
One day	-0.26 ± 0.13	-0.28 ± 0.13	0.336
One week	-0.18 ± 0.10	-0.20 ± 0.12	0.268
One month	-0.19 ± 0.08	-0.18 ± 0.10	0.502
Three months	-0.18 ± 0.08	-0.19 ± 0.09	0.469
IOP (mmHg)			
One day	19.56 ± 5.53	17.72 ± 4.37	0.018*
One week	17.31 ± 3.82	15.23 ± 3.19	<0.001*
One month	16.78 ± 3.46	16.25 ± 2.88	0.285
Three months	16.40 ± 3.59	16.17 ± 2.65	0.635

**Table 3:** The incidence of postoperative complications between the two groups AC: anterior chamber, aOR: adjusted odds ratio, CI: confidence interval * denotes a significant correlation to postoperative complications. The statistical significance was defined as P < 0.05.

Complications	Combined group	Single group	P
Ocular hypertension			
Incidence	12	16	-
Crude OR (95% CI)	1.22 (0.94-1.46)	Reference	0.097
aOR (95% CI)	1.10 (0.96-1.25)	Reference	0.203
AC inflammation			
Incidence	4	7	-
Crude OR (95% CI)	0.94 (0.85-1.04)	Reference	0.143
aOR (95% CI)	0.97 (0.92-1.02)	Reference	0.335

About the predisposing factor for ocular hypertension and AC inflammation in the combined group and single group, old age was correlated to a higher risk of developing ocular hypertension in the combined group (P < 0.001) and single group (P = 0.031) (Table 4). Besides, higher myopia is related to higher ocular hypertension risk in the combined group (P = 0.038) (Table 4). For the AC inflammation, the preoperative DED was associated with higher AC inflammation risk in the combined group (P = 0.001) and single group (P < 0.001) (Table 5). Also, high myopia is related to higher AC inflammation risk in the combined group (P = 0.044) and single group (P = 0.024).

**Table 4:** The predisposing factor for ocular hypertension in the two groups aOR: adjusted odds ratio, CI: confidence interval, DED: dry eye disease * denotes a significant correlation to ocular hypertension. The statistical significance was defined as P < 0.05.

Factor	aOR	95% CI		P
		Lower	Upper	
Combined group				
Old age	1.27	1.12	1.43	<0.001*
Male sex	1.12	0.95	1.31	0.115
Preoperative DED	1.02	0.94	1.11	0.528
Presence of systemic disease	1.09	0.93	1.24	0.429
High myopia	1.14	1.02	1.26	0.038*
Single group				
Old age	1.16	1.02	1.29	0.031*
Male sex	1.04	0.93	1.15	0.472
Preoperative DED	0.98	0.91	1.05	0.857
Presence of systemic disease	1.05	0.97	1.14	0.334
High myopia	1.08	0.98	1.17	0.086

**Table 5:** The predisposing factor for anterior chamber inflammation in the two groups aOR: adjusted odds ratio, CI: confidence interval, DED: dry eye disease * denotes a significant correlation to anterior uveitis. The statistical significance was defined as P < 0.05.

Factor	aOR	95% CI		P
		Lower	Upper	
Combined group				
Old age	1.03	0.94	1.13	0.465
Male sex	0.96	0.89	1.03	0.315
Preoperative DED	1.19	1.08	1.30	0.001*
Presence of systemic disease	1.11	0.92	1.32	0.346
High myopia	1.06	1.02	1.11	0.044*
Single group				
Old age	1.05	0.93	1.17	0.391
Male sex	0.97	0.90	1.04	0.758
Preoperative DED	1.22	1.10	1.35	<0.001*
Presence of systemic disease	1.14	0.91	1.36	0.289
High myopia	1.15	1.03	1.27	0.024*

## Discussion

Briefly, the combined usage of preoperative dexamethasone and postoperative prednisolone presented a similar anti-inflammatory effect compared to postoperative prednisolone monotherapy. Furthermore, the postoperative IOP was higher in the combined therapy than the prednisolone monotherapy in the early postoperative period. On the other side, the age, myopia degree, and preoperative DED status may influence the safety and efficiency of the two treatments.

In this study, the incidence of AC inflammation was similar between the combined and single groups after the cataract surgery. In the previous study, topical prednisolone is fair management to prevent or reduce the risk of post-cataract surgery inflammation [16]. On the other hand, the local dexamethasone implant also demonstrated excellent anti-inflammation ability after cataract surgery, which may be superior to topical prednisolone acetate [21]. Nevertheless, there was a scant study to evaluate whether the addition of topical dexamethasone on topical prednisolone is superior for controlling postoperative inflammation than the topical prednisolone monotherapy. To our knowledge, this research may be a preliminary experience to represent the similar anti-inflammatory effectiveness between prednisolone and dexamethasone combined treatment and prednisolone monotherapy. In addition, the baseline characteristics between the two groups were similar, which reduced the interference of initial status. Moreover, some confounders, such as age and sex, were adjusted in the multivariable analysis. As a consequence, the similar effectiveness between dexamethasone plus prednisolone therapy and prednisolone monotherapy in reducing post-cataract surgery inflammation may be credible. Prednisolone can effectively suppress inflammation in several ocular diseases and has been used for postoperative inflammation control for decades [22]. Because the individuals with inflammatory ocular disease like active uveitis or severe blepharitis were excluded and we kept the prednisolone therapy for one week in all the individuals, we speculate that the prednisolone monotherapy is adequate for those with mild preoperative ocular inflammation condition. Also, a low postoperative inflammation incidence of around 6% was found in both groups, which indicates the effectiveness of the two managements.

Concerning the safety of the combined treatment and single treatment, the incidence of ocular hypertension showed insignificant differences between the two groups. The usage of steroids may be the most common iatrogenic pathophysiology for ocular hypertension [23]. In a previous study, open-angle glaucoma can be induced by the persistent application of topical steroids [24]. In this study, the combined group did not demonstrate a significantly higher incidence of ocular hypertension compared to the single group, which may imply the usage of double steroid perioperatively does not cause significant risk compared to single steroid therapy. The incidence of ocular hypertension between the two groups was 19.4% and 14.8%, and no IOP higher than 21 mmHg was recorded one month postoperatively, which indicates the IOP elevation could be transient after the usage of combined or single therapies in this study. However, the mean IOP value was significantly higher in the combined group compared to the single group one day postoperatively and one week postoperatively. The results may indicate that the usage of steroids for a longer time still contributes to the higher risk of ocular hypertension, although the total time of high-potency steroid administration was two weeks. The mean IOP value in the combined group did not reach the threshold of ocular hypertension and was reduced to a similar value as the combined group one month postoperatively. The above findings suggest that the elevated IOP induced by combined treatment was not persistent but may cause some damage in patients with pre-existing glaucoma. As a consequence, the patients with glaucoma may not be suitable for the combined dexamethasone and prednisolone therapy. An alternative anti-inflammatory agent, such as ketorolac [15], may be considered in such patients.

Regarding the predisposing factor for ocular hypertension and AC inflammation in different groups, old age is associated with a higher risk of postoperative ocular hypertension in both the combined group and single group. The old age is a known risk factor for the ocular hypertension and glaucoma [25]. In a previous study, the development of glaucoma was positively correlated to age, in which the incidence of glaucoma was 0.5% in people aged 30-39 years and 6.1% in people aged older than 70 years [26]. As a result, it may be reasonable that the patients with old age were at higher risk of ocular hypertension regardless of the type of anti-inflammatory management in this study. Except for old age, the high myopia was related to a higher incidence of ocular hypertension in the combined group but not the single group. The high myopia status was associated with a higher risk of IOP elevation in the previous study [27], and glaucoma also develops in the high myopia population more frequently [28]. The findings in this study may further indicate that the combination of dexamethasone and prednisolone applications may induce ocular hypertension in the vulnerable population. On the other side, high myopia and preoperative DED status correlated to the higher possibility of AC inflammation in both the combined group and the single group. High myopia is accompanied by higher inflammation status in the general population [29], and DED is an inflammatory ocular surface disease that features elevated interleukin levels [30]. Our results corresponded to the conclusion in previous literature.

There were several limitations in this study. Firstly, the retrospective design of this study would reduce the homogeneity of the study population despite the fact that the initial characteristics did not present a significant difference between the two groups. Secondly, we did not match the eye numbers between groups, and there was a discordance of eye numbers between the combined group and the single group. Besides, we did not measure the postoperative DED parameter or the meibomian gland dysfunction status due to the retrospective design, and both parameters may provide a better explanation for the AC inflammation control between groups. Finally, all the patients in this study were Han Taiwanese, and the external validity of this study may be relatively low.

## Conclusions

In conclusion, the usage of perioperative dexamethasone and prednisolone revealed similar postoperative inflammation control and IOP elevation compared to prednisolone monotherapy. Furthermore, the patients with high myopia correlate to a higher risk of ocular hypertension in the combined treatment but not the single treatment. Consequently, prednisolone monotherapy may be adequate to control postoperative inflammation with fair safety for the general population. Further large-scale prospective study to evaluate the anti-inflammatory effect of combined treatment and prednisolone monotherapy in individuals vulnerable to ocular inflammation is mandatory.
